# Stem Cell Research Tools in Human Metabolic Disorders: An Overview

**DOI:** 10.3390/cells10102681

**Published:** 2021-10-07

**Authors:** Serena Ricci, Pietro Cacialli

**Affiliations:** 1 Department of Cell Physiology and Metabolism, School of Medicine, University of Geneva, Rue Michel Servet 1, 1206 Geneva, Switzerland; serena.ricci@unige.ch; 2 Department of Pathology and Immunology, School of Medicine, University of Geneva, Rue Michel Servet 1, 1206 Geneva, Switzerland

**Keywords:** stem cell therapy, diabetes, obesity, lysosomal storage diseases

## Abstract

Metabolic disorders are very common in the population worldwide and are among the diseases with the highest health utilization and costs per person. Despite the ongoing efforts to develop new treatments, currently, for many of these disorders, there are no approved therapies, resulting in a huge economic hit and tension for society. In this review, we recapitulate the recent advancements in stem cell (gene) therapy as potential tools for the long-term treatment of both inherited (lysosomal storage diseases) and acquired (diabetes mellitus, obesity) metabolic disorders, focusing on the main promising results observed in human patients and discussing the critical hurdles preventing the definitive jump of this approach from the bench to the clinic.

## 1. Introduction

Metabolic disorders usually occur as a result of loss and/or deficiency of enzymes or hormones indispensable for converting one metabolite to another metabolite [1]. Disorders of metabolism are very common in the population worldwide [2] and are among the diseases with the highest health utilization and costs per person [3]. Generally, these disorders are classified into two main groups: acquired and inherited [4]. Acquired metabolic diseases, such as diabetes mellitus or obesity, are linked to different external factors, such as an unhealthy lifestyle along with reduced physical exercise and high caloric intake [4]. Differently, inherited metabolic disorders, such as lysosomal storage diseases (LSDs), and peroxisomal and metal metabolism diseases, are caused by inborn errors of metabolism resulting from genetic defects [4]. While clinical trials are in progress for possible treatments for some of these diseases, there is currently no approved therapy for many of them, resulting in a huge economic hit and tension for society [5]. In this review, we recapitulate the recent advancements in stem cell and gene therapy as new potential tools in treating metabolic diseases, focusing on diabetes, obesity and LSDs, which account for the largest share of the global market of therapies for metabolic disorders. In particular, we highlight the main promising results observed in human patients, underlining the critical hurdles preventing the use of these methodologies in routine clinical practice.

### 1.1. Diabetes Mellitus

**Figure 1:**
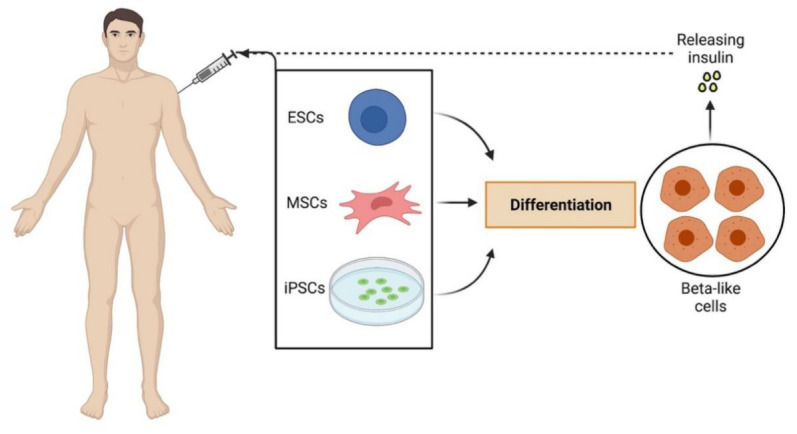
Representation of main stem cell approaches in type 1 and type 2 diabetes. Abbreviations: ESCs: embryonic stem cells; MSCs: mesenchymal stem cells; iPSCs: induced pluripotent stem cells.

Diabetes mellitus (DM) represents one of the most critical and costly health issues affecting millions of people worldwide and contributing to 5% of global deaths in young people [6,7]. According to the most recent global report of the World Health Organization, the incidence of diabetes has strongly increased in recent years, especially in low and middle-income countries, with the number of people affected rising from 108 million in 1980 to 422 million in 2014, and an estimated 1.5 million deaths in 2012 directly caused by the disease (ISBN: 9789241565257, https://apps.who.int/iris/handle/10665/204871 accessed on 30 August 2021). Low-efficient pharmacological therapies together with poor patient compliance in the application of adequate life regimes strongly contribute to the increasing incidence of disease-related complications, which economically impacts countries’ health systems [8]. Thus, a major challenge in diabetes research is the identification of innovative therapeutic, or even curative, tools able to work at a group level to the extent of stopping the disease both at its early and late stages. In recent years, the stem cell strategy for curing DM has been arising great enthusiasm, as the approach offers the theoretical advantage of achieving a durable and definitive benefit by a single or a few treatments [9]. The pre-clinical data generated on animal models and the successful applications in human clinical trials deserve attention as potential curative tools for this disease (Figure 1).

### 1.2. Type 1 Diabetes (T1D)

T1D is a chronic disease of childhood and young adults that affects at least 30 million people worldwide, with an increasing incidence of 2–3% per year [10]. Despite the fact that in humans, many issues related to the immunological nature of T1D are still to be solved [11], it is widely accepted that the disease derives from an autoimmune destruction of insulin-producing β cells, resulting in insulin deficiency and hyperglycemia, necessitating lifelong insulin replacement [12]. Despite the fact that genome-wide association studies identified that approximately 50% of the genetic risk of the disease is attributable to human leukocyte antigens [13], the increasing incidence of T1D in recent decades has defined important implications of environmental/behavioral factors (e.g., infant diet, viruses, decreased gut microbiome diversity) [14]. Different animal models such as non-human primates [15,16], large animals (dog and pig) [17], rodents [18] and, recently, the non-mammalian zebrafish model (for its regenerative properties in different tissues [19]) [20,21] have been used to study the causes and complications of T1D. However, the exact causes of T1D are still poorly understood. The main symptoms ascribed to T1D are excessive hunger, excessive thirst, blurred vision, fatigue, frequent urination, weight loss in a short period of time and a dramatic complication of diabetes, known as ketoacidosis [22,23]. This complication includes rapid breathing, dry skin and mouth, flushed face, fruity breath odor, nausea, vomiting or stomach pain [24,25]. Tools for managing T1D continue to improve, although insulin remains the main therapy which, unfortunately, does not always achieve the correct metabolic control and cannot impede the occurrence of complications, requiring strict monitoring, education of patients and continuous insulin dose adjustments [26]. Furthermore, it has been shown that around 25.5% of patients with T1D can develop obesity and insulin resistance (“double diabetes”) [27]. This means the insulin they obtain from injections does not work as well as it should. For these patients, in addition to insulin therapy, the use of metformin, an oral medication commonly used in T2D management, has been approved, and it has been shown to reduce insulin dose requirements, although the appropriateness of its use is still controversial [28,29].

### 1.3. Current Advances in Stem Cell-Based Therapies for T1D

In recent years, big expectations have been placed on beta cell regeneration by the stem cell strategy as a promising cure for T1D [30]. The first enthusiastic results were obtained with the use of pluripotent stem cells (PSCs), including human embryonic stem cells (hESCs) and induced PSCs (iPSCs), which represent an unlimited supply of bona fide insulin-producing cells for autologous/allogenic transplantation [31]. Recent improvements in the PSC-derived beta cell microenvironment (e.g., microencapsulation) have allowed reducing the immune rejection of grafts and increasing the surface area for nutrients and oxygen to avoid ischemia [32]. However, the remaining challenges still need to be addressed regarding how to deliver PSC cells without great loss, without immune suppression and re-occurring autoimmunity and, in particular, with sufficient safety, in order to allow widespread implementation of PSC therapy for T1D patients [33]. Differently, the use of hematopoietic stem cells (HSCs) has recently been considered the most promising approach because of the differentiation and expansion capabilities of these cells, which ensure that a gene added to a small number of cells results in gene correction of much greater numbers of differentiated cells circulating in the whole organism [34,35,36]. Specifically, in curing type 1 diabetes, several studies in newly diagnosed T1D patients showed the powerful ability of autologous HSC transplantation to modulate T cell proliferation and pro-inflammatory cytokine production [37]. The immunomodulation reduces the aggressive autoimmune destruction of beta islets and has been observed to partially repristinate pancreas functionality and glycemic control [38]. However, the majority of patients who underwent transplantation were not able to achieve a long-lasting lowering of insulin dependence and prevention from its complications [39]. Finally, several studies have also explored the efficacy of the use of adult tissue-derived mesenchymal stem cells (MSCs) in T1D [40,41]. These studies reported the safety and efficacy of co-infusion of insulin-secreting adipose-derived MSCs and bone marrow-derived HSCs in patients with T1D, in which the use of an autologous inoculum appeared to confer better long-term control of hyperglycemia compared to allogenic stem cell transplantation [41]. The therapeutic property of MSCs has been revealed to be able to preserve the remaining existing beta cells, through immunomodulation, as well as giving rise to new functional ones [42]. Despite the vast number of sources of MSCs (bone marrow, adipose tissue, umbilical cord, etc.) and hypo-immunogenicity [43], data relative to their ability to differentiate into efficient beta cells remain controversial, and the doubt concerning spontaneous tumorigenicity capacities [44] still impedes the translation of this tool from the bench to beyond.

### 1.4. Type 2 Diabetes (T2D)

T2D represents the most common form of DM of great concern in global healthcare. In 2017, it was estimated that around 6.28% of the world’s population was affected by T2D, and, since then, over 1 million deaths per year have been attributed to this disease, making it the ninth leading cause of global mortality [45]. Multiple disturbances including impaired insulin secretion and insulin resistance in the main insulin-responding tissues (muscle, liver and adipocytes) contribute to the etiopathogenesis of T2D, resulting in abnormal glucose homeostasis [46]. Treatment of T2D remains mainly focused on ameliorating metabolic controls and on reducing the risk of acute and chronic complications (e.g., blindness, nerve damage, heart attack, stroke) [47]. In combination with improvements in patients’ lifestyle (physical activity, diet, etc.), the main therapeutic approach for patients with T2D is represented by metformin administration [48]. Metformin is able to reduce the hepatic glucose output, to enhance peripheral tissue insulin sensitivity and to stimulate GLP-1 secretion [49]. However, many other adjunctive/combined drugs have shown valuable efficacy in the management of the disease such as sulfonylureas, GLP-1 receptor agonists, SGLT-2 inhibitors and, recently, thiazolidinediones [47].

### 1.5. Stem Cell Therapy Approach to T2D

Stem cells have also been considered a highly promising means for the treatment of T2D due to the fact that, as in T1D, patients affected show an injured pancreas and impaired insulin production, especially in the late stage of the disease [50]. Stem cell administration indeed showed important immunomodulatory properties and a regenerative ability to stimulate the recovery of injured pancreases [51]. Different studies reported the generation of interspecific pancreatic chimeras from pancreatic stem cells [52] and the rat pancreas in mice [53], pigs [54] and rhesus monkeys [55]. While few pre-clinical reports have outlined the effectiveness of the use of iPSCs in T2D animal models [56,57], the majority of human studies, which found an encouraging applicability of this tool in the clinic, focused on the autologous administration of MSCs [58]. Pre-clinical data elucidated, indeed, that MSCs are not only able to differentiate in functional insulin-producing cells but are capable of preserving the integrity of the remaining beta cells and, overall, of promoting insulin sensitivity in peripheral tissue, activating insulin receptors and acting on macrophages’ inflammatory status [58]. In humans, transplantation of bone marrow-derived MSCs was observed to be safe and to have good tolerability, with a successful ability to not only improve metabolic parameters (e.g., glycemia, HbAc1, body weight, fat mass) but also to ameliorate disease-related complications [59,60]. Interestingly, patients who underwent treatment showed a lowering in insulin requirements, sustained in the long term, with improvements in insulin sensitivity [61,62]. Nevertheless, the great heterogenicity in the single patient clinical picture does not allow drawing a defined phase of the disease that the tools can be applicable in [63]. Moreover, the route of administration (targeted or peripheral) can have an important impact on the outcome, and thus it represents a limiting factor [64]. Finally, in spite of the tested safety, patients should remain under close surveillance for checking the eventual tumorigenicity potential of delivered cells [64].

### 1.6. Obesity

With more than 650 million people affected worldwide, obesity represents one of the most devastating health issues of Western industrialized countries [65]. The latest WHO report observed an increasing prevalence of this disorder that nearly tripled between 1975 and 2016, with a dramatic growing incidence in the pediatric age—more than 30 million children up to the age of 5 years old are obese (https://www.who.int/news-room/fact-sheets/detail/obesity-and-overweight accessed on 30 August 2021). The disease, broadly defined as an excess of body fat mass (body mass index (BMI) more than 30 kg/m^2^), has a complex etiology with a combination of genetic, hormonal and environmental factors [66]. Following the most recent recommendations for the non-pharmacological management of obesity, the main therapeutic approach for affected patients is diet therapy, physical activity and behavioral therapy [67]. However, for a great percentage of patients, initial success in weight loss can hardly be maintained in the longer term without constant vigilance to sustain behavior changes in the face of environmental pressures to regain weight [68]. This is why adjunctive drug therapies (statins, anorectics, anti-depressives, GLP-1 agonists, etc.) are required to help patients in sustaining sufficient weight loss and prevent obesity-associated complications (diabetes, vascular disease, hypertension, fatty liver, etc.) [69].

### 1.7. Stem Cell Therapy to Repristinate Metabolic Homeostasis in Obese Patients

The possibility to apply stem cell therapy in the treatment of obesity arose after a pre-clinical study observed that transplantation of adipose tissue from wild-type mice into *ob/ob* mice was able to repristinate metabolic homeostasis [70,71]. Some years later, these results found explanation in the phenomenon of adipose dysfunction characterizing obesity [72]. Patients with obesity show, indeed, an impaired adipose tissue function with adipocyte hypertrophy, hypoxia and inflammation which alter the cellular composition, increase lipid storage and impair insulin sensitivity [73]. The first attempts of stem cell therapy were, thus, intended to improve fat dysfunction by autologous transplantation of adipose-specific mesenchymal stem cells (called adipose-derived stem cells—ASCs), a population of multipotent cells with the ability to differentiate into various cell lineages [74]. Infusion of ASCs in high-fat diet-mediated obese mice showed a great improvement in body weight, glucose tolerance and dyslipidemia with a valuable reduction in fat tissue hyperplasia [75,76,77]. Despite the fact that no clinical study applying ASCs to treat obese patients has been started yet, the enthusiastic pre-clinical data obtained encourage the translation of this approach in humans, also considering the great availability of ASC sources [78]. In obese subjects, it has been shown, indeed, that minimally invasive and low-morbidity liposuction of subcutaneous/visceral fat allows isolation of an incredible amount of ASCs, sufficient for autologous transplantation [78]. Another interesting aspect related to stem cell application in the treatment of obesity is the potential of ex vivo differentiation into brown adipocytes, which is reported to improve obesity in animals and humans [79,80,81,82]. In this case, interesting results were obtained by the use of induced pluripotent stem cells (iPSCs) which can be easily collected and engineered ex vivo for being retransplanted into the original donor [83]. Transplantation of induced brown adipocytes (iBAs)—differentiated from epidermal-derived iPSCs—showed a strong ability to ameliorate the obese phenotype, by reducing body weight and ameliorating glucose tolerance and dyslipidemia [84]. In spite of these promising observations, transplantation in humans of ex vivo iBAs still seems to be a far-off goal, due to the difficulties in mimicking the fat physiological microenvironment. It has been observed, indeed, that highly vascularized lobular structures within adipose tissue correspond to the key environment where the browning phenomenon occurs and which cannot be re-produced in vitro [84,85].

Finally, a real challenging attempt to adopt stem cells to treat obesity has been conducted with the use of neural stem cells (NSCs). The role of the central nervous system in controlling appetite and feeding behavior [86,87] is widely recognized, and particularly very well described is the dense network between hypothalamic and non-hypothalamic hormones—but which acts at the hypothalamus levels—having a determined impact in the pathophysiology of obesity [87]. Recent evidence outlined that impaired food intake observed in obesity can be due to the inflammation-mediated loss of neural stem cells in the hypothalamus which affects the regeneration of neurons in this particular brain area [88,89]. In mice, it was shown that virus-mediated disruption of hypothalamic NSCs is able to induce glucose intolerance and to affect body weight [90], as well as knocking out the leptin receptor from these cells, which promotes an extreme obese phenotype [91]. However, realizing a concrete attempt to translate these studies in patients requires overcoming the strong controversy persisting in the scientific community about the existence of NSCs in the human brain [92,93].

### 1.8. Lysosomal Storage Diseases (LSDs)

Lysosomal storage diseases (LSDs) encompass more than 70 inherited diseases, which occur during infancy and childhood (1:5000 newborns), mainly characterized by a progressive neurodegenerative clinical course [94]. Despite the fact that the pathogenesis is still not currently well understood, the main mechanism causing the disease is the accumulation of metabolic substrates within lysosomes or peroxisomes, due to genetic defects in lysosomal enzymes or lysosomal membrane proteins [95,96,97]. Lysosomal dysfunction affects, basically, all body tissues, although its serious effects mainly concern the central nervous system, meaning that affected patients display progressive psycho-neurological impairments, accompanied by a broad spectrum of other clinical phenotypes, such as seizures, facial and other bone deformities, problems with vision and hearing, difficulty breathing, anemia and an enlarged spleen or liver [98,99]. For many LSDs, there are approved disease-specific therapies based on enzyme replacement (ERT) [100,101,102], or on intravenous administration of recombinant proteins [103,104,105], accompanied by treatment of the neurological complications, such as anticonvulsant medication, ventilatory support, assistance for patients with learning disability, orthopedic interventions and nutritional support [106,107]. Despite the good tolerability demonstrated by ERT, unfortunately, many symptoms, even after long-term treatment, are not reversible [108]. In addition, many patients showed development of antibodies against the exogenous enzyme/protein with a negative impact on the therapy efficacy [109]. This is particularly true for LSDs severely affecting the CNS [110,111,112]. New therapeutic drugs for several LSDs, including substrate inhibitors and chaperones, have been approved considering their ability to overcome ERT’s limitations; still, their efficacy seems to be variable among patients [113,114].

### 1.9. Treating LSDs by Stem Cell Gene Therapy

The approach of using stem cells for treating LSDs soon appeared as very challenging, but the promising advanced pre-clinical results have allowed many of these attempts to progress into clinical experimentation [115]. In particular, the use of stem cells has been referred to as a means for gene defect correction [115].

**Figure 2:**
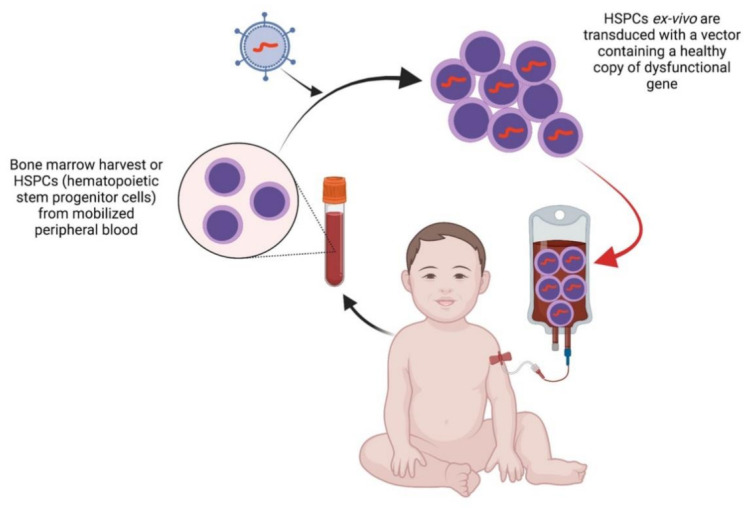
Representation of the main stem cell gene therapy approach for treating lysosomal storage diseases. Abbreviations: HSPCs: hematopoietic stem progenitor cells.

Several studies in animal models of different types of LSDs indeed showed that genetic editing of autologous/allogenic blood stem progenitors (HSCs) and their transplantation can ensure increased production of the missing lysosomal enzyme or protein [116,117,118,119], with partial improvements in the phenotypical features of the disease [120,121,122]. The rationale to apply HSC transplantation in LSDs lies in the ability of the transplanted cells and/or their progeny to contribute to fixed tissue macrophage populations in the affected tissues and to become local permanent sources of functional lysosomal enzymes [123,124,125] (Figure 2). Of note, interesting is the recent pre-clinical evidence in the stem cell approach to Pompe disease, where transplantation in mice of bone marrow-derived HSCs transduced by a highly efficient gene transfer method (a third-generation SIN-lentiviral vector containing a codon-optimized transgene driven by the strong SFFV promoter) resulted in high and sustained enzyme activity in the tissues that are most affected in Pompe disease [126].

In humans, the first trials, around 25 years ago, showed that in patients with Gaucher disease (lacking the functional glucocerebrosidase enzyme in macrophage cells), the autologous transplantation of viral-mediated modified HSCs (peripheral blood or bone marrow-derived) encoding the normal enzyme allows the amelioration of enzyme deficiency [127,128]. Some years later, a similar approach was tested in an animal model and three patients with neuronal ceroid lipofuscinosis (NCL) (lacking the functional palmitoyl protein thioesterase 1—PPT1) by transplanting them with genetically engineered bone marrow-derived HSCs overexpressing PPT1 [129]. However, PPT1 enzyme activity was normalized only in peripheral leukocytes with a mild amelioration of the clinic manifestations [130]. Functional improvements in cognitive and orthopedic features were observed to be long-lasting in patients affected by Hurler syndrome (deficient in lysosomal alpha-L-iduronidase activity) and transplanted with engineered peripheral blood-derived HSCs [131]. In addition, poor achievements with the first HSC transplantation can sometimes be improved by a second transplantation, which has shown good tolerance and clinical efficacy [132]. The approach of using modified peripheral blood-derived HSCs in pediatric patients affected by Faber disease (*N*-acylsphingosine amidohydrolase deficiency) provided an enthusiastic resolution of clinical manifestations, and patients survived for decades with no more complications [133,134]. Instead, peripheral blood-derived HSC delivery therapy in 17 patients affected by alpha-mannosidosis seemed to be less successful, as patients made scant developmental progress [135]. Still, amazing results were obtained from autologous infusion of gene-corrected peripheral blood-derived HSCs in patients affected by metachromatic leukodystrophy, for whom the disease stopped progressing in the long term [136,137].

Some challenging attempts at stem cell therapy application have also been conducted by the use of neuronal stem cell progenitors (NSCPs). Allogenic transplantation of NSCPs from healthy donors in newborn mice affected by mucopolysaccharidosis VII showed restoration of normal neural cell function [138,139]. Similarly, transplantation of NPCs genetically modified to overexpress the human beta-hexosaminidase alpha-subunit gene in mouse models of Tay-Sachs disease resulted in amelioration of neurological features with potential therapeutical insights [140]. Still, transplantation of genetically modified NPCs in mouse models of Niemann–Pick disease (deficiency in acid sphingomyelinase—ASM) seems to be sufficient to reverse lysosomal storage pathology [141]. Impressing results were also obtained by NPC transplantation in mouse models of NCL for repristinating PPT1 expression and neuroprotection [142].

Transplantation of genetically modified stem cells directly into the brain was also tested by the use of embryonic stem cells (ESCs). In guinea pig models of alpha-mannosidosis (alpha-d-mannosidase enzyme deficiency), modified ESCs were successfully implanted [143]; however, uncontrolled cell proliferation and formation of brain teratomas drew the unfeasibility in the use of this pluripotent cell type for achieving longer-term disease treatment [143]. On the contrary, some years later, in a mouse model of Sanfilippo syndrome (mucopolysaccharidosis IIIA), intracerebral transplantation of sulfamidase-expressing ESCs was observed to be safe [144].

Further studies, in recent years, have also shown the possibility to easily obtain autologous stem cells by non-invasive methods, and in a greater volume, by the use of induced pluripotent stem cells (iPSCs) [145]. Pre-clinical evidence in animal models of LSDs showed that brain administration of corrected iPSC-derived neuronal stem precursors was able to reverse the neuropathology in a zone surrounding the grafts [146,147]; however, their application in humans still seems to be far away [148]. In Supplemental Table S1, we report all recent stem cell (gene) therapy applications in animal models and/or human patients for different metabolic disorders.

## 2. Conclusions and Perspectives

In conclusion, despite the fact that greater efforts are still needed for solving the remaining issues hampering the translation of the stem cell approach into the clinical routine for the management of type 1 and type 2 diabetes patients, the promising achievements obtained until now give us hope and solid expectations that a disease, until now considered “uncurable”, could be definitively cured in the near future. Despite this, envisioning the application of cell therapy in such a multifactorial disease as obesity remains challenging; however, history has taught that scientific potential and improvements in technology can turn a far-off goal into a powerful reality. Differently, it is possible to assess that stem cell-based therapy in the management of LSDs has been revealed as the most promising and effective tool in the long term for curing these diseases. In perspective, further studies are surely needed in order to define standardized protocols for clinical application of stem cells in metabolic disorders. However, the research efforts until now and those ongoing encourage the medico-scientific community in tackling the great step head which can be conducted with these new therapies to significantly improve patients’ life quality and prolong their survival.

## Data Availability

Not applicable.

## Supplementary Materials

The following are available online at https://www.mdpi.com/article/10.3390/cells10102681/s1, Table S1: Landmark reports of stem cell (gene) therapy application in animal models and/or human patients for the different metabolic disorders.
