# Supplementary material for: Stem Cell Research Tools in Human Metabolic Disorders: An Overview
Source: Cells. 2021 Oct 7;10(10):2681. doi: 10.3390/cells10102681 (PMC8534517; doi:10.3390/cells10102681)
Supplement: Supplementary file 1 [file cells-10-02681-s001.zip › cells-1385010-supplementary.pdf]

	Stem Cell type	Landmark reports
Type 1 Diabetes	HSCs	Voltarelli J.C. *et al.*, 2007<br>D'Addio F., 2014<br>Ye L. *et al.*, 2017
	MSCs	Cai J. *et al.*, 2016<br>Ulyanova O. *et al.*, 2019
Type 2 Diabetes	iPSCs	Alipio Z. *et al.*, 2010
	MSCs	Bhansali A. *et al.*, 2014<br>Bhansali S. *et al.*, 2017<br>Al Demour S. *et al.*, 2018<br>Chen Y. *et al.*, 2018
Obesity	ASCs	Yoshimura K. *et al.*, 2006<br>Cao M. *et al.*, 2015<br>Lee C.W. *et al.*, 2017
	iPSCs	Kishida T. *et al.*, 2015
	NSCs	Li J. *et al.*, 2014<br>Ren Z. *et al.*, 2020
Lysosomal Storage Diseases	HSCs	Dunbar C. et al., 1996<br>Lönnqvist T. et al., 2001<br>Biffi A. *et al.*, 2006<br>Yoshimitsu M. *et al.*, 2007<br>Enquist I.B. *et al.*, 2009<br>van Til N.P. *et al.*, 2010<br>Mynarek M. *et al.*, 2012<br>Biffi A. *et al.*, 2013<br>Sessa M. *et al.*, 2016
	NSCs	Snyder E. Y. *et al.*, 1995<br>Lacorazza H.D. *et al.*, 1996<br>Buchet D. *et al.*, 2002<br>Meng X. L. *et al.*, 2003<br>Shihabuddin L. S. *et al.*, 2004<br>Sidman R. L. *et al.*, 2007
	iPSCs	Doerr J. *et al.*, 2015<br>Griffin, T. A. *et al.*, 2015<br>Meneghini V. *et al.*, 2017<br>Kobolak J. *et al.*, 2019<br>Clarke D. *et al.*, 2018

Supplemental Table 1. Landmark reports of stem cell (gene) therapy application in animal models and/or human patients for the different metabolic disorders. Abbreviations: iPSCs: induced Pluripotent Stem Cells; MSCs: Mesenchymal Stem Cells; ESCs: Embryonic Stem Cells; HSCs: Hematopoietic Stem Cells; ASCs: Adipose-derived mesenchymal Stem Cells; NSCs: Neuronal Stem Cells
